# Nurses’ experiences with assessing pain in patients with dementia in nursing homes: a qualitative study

**DOI:** 10.1186/s12912-026-05110-z

**Published:** 2026-07-27

**Authors:** Arlén Akselberg, Oda Dyrnes Schei, Stine Hauvik

**Affiliations:** 1 Department of Emergency Medicine, Ambulance Team, Helse Bergen, Bergen, Norway; 2 Skincare Clinic, Din Hud V/Oda Dyrnes Schei, Surnadal, Norway; 3 Department of Health Sciences and Social Care, Molde University College, Britvegen 2, Molde, 6410 Norway

**Keywords:** Dementia, Experience, Nursing, Nursing home, Pain assessment

## Abstract

**Background:**

Approximately 80% of long-term care residents in Norwegian nursing homes have dementia. Patients with dementia represent a vulnerable group that tends to underreport pain, mainly due to difficulty expressing pain. Nurses are responsible for assessing pain in patients with dementia when signs of discomfort are evident through behavioural changes. Knowledge of the diagnosis and the patient is essential for proper detection and assessment. We aimed to explore nurses’ experiences in assessing pain in patients with dementia in nursing homes.

**Methods:**

Data were collected through eight individual interviews with nurses at two Norwegian nursing homes in May 2024. To analyse the data, a qualitative design using thematic analysis was conducted following Braun and Clarke’s six steps.

**Results:**

Three main themes were identified through analysis: (1) pain assessment is similar to detective work; (2) pain assessment of patients with dementia requires collaboration; and (3) nurses’ desire to improve the quality of pain assessment for patients with dementia.

**Conclusion:**

Pain assessment in people with dementia was described by nurses as a complex process requiring the interpretation of behavioural changes, knowledge of the individual patient, and the integration of information from multiple sources. The findings highlight the importance of clinical reasoning, collaboration, and continuity in supporting pain assessment when self-reports are limited.

**Supplementary Information:**

The online version contains supplementary material available at https://doi.org/10.1186/s12912-026-05110-z.

## Introduction

Dementia is a public health priority worldwide because of its rapidly increasing prevalence and the challenges associated with providing proper care [1]. Approximately 50 million people live with dementia worldwide, and approximately 115,000 people are diagnosed in Norway. An estimated 80% of long-term care residents in Norwegian nursing homes have dementia, with the prevalence of dementia expected to rise as the population ages [2].

The term dementia encompasses various diseases that gradually damage the brain. It leads to memory loss, disorientation, language and communication difficulties, and instability in various areas, such as emotions, socialisation, and initiative [3]. Due to these impairments, individuals with dementia, particularly those in severe stages, often have difficulty expressing pain and are prone to underreporting it. This group might struggle to articulate sensations and instead exhibit symptoms, such as restlessness, fear, or agitation [4, 5].

With advancing age, the incidence of pain increases significantly, making it one of the most common and burdensome symptoms in older adults. Studies estimate that up to 80% of nursing home residents experience daily pain [6, 7]. This is especially concerning given that pain in older adults, particularly those with cognitive impairments, such as dementia, is often underreported and undertreated [8]. Persistent, untreated pain can severely affect well-being, impacting sleep, appetite, mobility, and quality of life. Moreover, it is linked to increased risks of depression, anxiety, social withdrawal, and functional decline [9, 10], and may increase fall risk, exacerbating frailty and dependence. Research underscores that pain in older adults is a multidimensional experience influenced not only by physical factors, but also by psychological, social, and existential factors [11, 12].

Patients with dementia tend to underreport pain due to challenges in communication, memory, and sensory interpretation [13–15]. Consequently, their pain is often inadequately assessed and insufficiently treated [16–18]. Norwegian national guidelines stress the importance of recognising neuropsychiatric symptoms (NPS), such as agitation, restlessness, or withdrawal, as potential pain indicators in patients with dementia, and recommending systematic assessment and, when appropriate, treatment as pain related [3].

Nearly all patients with dementia experience NPS during the course of the disease. To distinguish between NPS caused by the progression of dementia and those caused by pain, nurses and other healthcare personnel must employ an evidence-informed, standardized approach that integrates pharmacological and non-pharmacological strategies. The Targeted Interdisciplinary Model for Evaluation and Treatment of Neuropsychiatric Symptoms (TIME) is a multicomponent, evidence-based intervention grounded in principles of cognitive behavioural therapy [19] Within the TIME intervention, person-centred assessment is guided by the VIPS framework [20], which places the person’s individual needs, daily experiences, and perspectives at the centre of clinical assessment and care planning. This approach helps interdisciplinary teams better understand the underlying causes of NPS and tailor interventions accordingly.

Over the years, several studies on pain in patients with dementia have focused predominantly on aspects such as pain location, intensity, and other pain-related characteristics. Within the Norwegian context, several researchers have developed and tested observation-based tools, such as the Doloplus-2 [21–22], Mobilization–Observation–Behaviour–Intensity–Dementia (MOBID-2) [23], and the Checklist of Nonverbal Pain Indicators (CNPI) [24] for assessing pain in patients with dementia. However, effective assessment, understanding and management of pain in this patient population are contingent upon the initial recognition and identification of symptoms related to pain [16, 18, 25].

Healthcare professionals often face significant challenges in pain identification, relying on nonverbal cues and behavioral changes, highlighting the need for personalised, context-sensitive assessments and improved staff training [26]. Research from Spichiger and colleagues [27] showed that nurse-led interventions using repeated case studies in nursing homes increased pain-free intervals and reduced pain frequency among patients with dementia, although pain intensity remained difficult to measure [27].

Institutional constraints, combined with a feeling of uncertainty and nurses’ own lack of confidence, often hinder effective pain management for patients with dementia. These findings underscore the need for nuanced, interdisciplinary approaches that integrate clinical observation, caregiver input, and patient history to avoid overlooking or misinterpreting pain [16, 28]. Given the challenges that patients with dementia frequently face in verbally communicating their pain, it is essential to investigate nurses’ experiences in identifying nonverbal and behavioral expressions that can indicate pain in a nursing home setting. Few studies have explored nurses’ experiences with the assessment process itself. Therefore, the aim of this study was to explore nurses’ experiences in assessing pain in patients with dementia in nursing homes.

## Materials and methods

### Design

A qualitative, interpretive, and descriptive design was employed to explore the topic, drawing on participants’ perspectives and experiences to capture diverse understandings and gain in-depth knowledge [29] of pain assessment in patients with dementia in nursing homes. The study was informed by an interpretivist paradigm, which recognises that knowledge is socially constructed and that individuals’ experiences provide valuable insights [30] into how pain assessment is understood and enacted in clinical practice.

### Study setting, recruitment, and participants

The researchers submitted a formal application and invitations to participate in the study to two nursing homes in different municipalities in Western Norway, which served as the study’s research settings. After approval, nursing home management forwarded the invitations to eligible nurses and included information about the study aim, a project description, a consent form, and an interview guide.

The study’s inclusion criteria required that the participants were (1) registered nurses working in nursing, (2) had experience caring for patients with dementia, and (3) were involved in pain assessment as part of their clinical practice. The participants were also required to (4) be able to communicate verbally in Norwegian and (5) provide informed consent. Nurses without experience caring for patients with dementia or conducting pain assessments in this patient group were excluded from participation in this study.

Twenty-two nurses working with patients with dementia in two nursing homes were eligible and therefore invited to participate. Fourteen nurses did not respond to the invitation to participate in the study. As a result, six interviews were conducted with nurses at one nursing home, whereas only two were conducted with nurses at the other. Both nursing homes were long-term care facilities for patients requiring continuous, 24-hour healthcare services. The interviews were scheduled at the participants’ workplaces with nurses who agreed to participate. In total, eight semistructured interviews were conducted. The nurses’ experience ranged from 1.5 to 33 years; all the nurses were women and had experience working in dementia care in nursing homes.

### Data collection

An interview guide (supplementary file 1) was developed and pilot-tested by the first and second authors to ensure alignment with the study aim. The questions were open-ended and flexible in sequence, allowing the interviewer to explore emerging themes raised by participants [29].

Individual semistructured interviews were conducted in May 2024 by the first and second authors. To establish trust, the interviewers introduced themselves, explained their interest in the topic, and outlined their reasons for conducting the research. The interview guide included broad questions, such as *‘Can you tell us about your experiences assessing pain among nursing home patients with dementia?’* with follow-up prompts such as, *‘Can you explain your experiences in more detail?’* In reflexive thematic analysis, sample adequacy is determined by the richness of the data rather than by achieving data saturation. The authors considered a sample of eight participants sufficient because the participants provided information-rich accounts that offered substantial depth and complexity, enabling a rigorous and nuanced interpretation of the phenomenon [31]. The interviews lasted approximately 45–60 min, were audio-recorded, and were transcribed verbatim. The transcripts were not returned to the participants for review, as the researchers sought clarification and feedback during the interviews themselves.

### Data analysis

Data were analysed using reflexive thematic analysis as outlined by Braun and Clarke [32]. This flexible qualitative approach was employed to identify patterns of meaning within the dataset. Following the six phases of thematic analysis, the first and second authors read each transcript several times to familiarise themselves with the data and gain an overall understanding of the participants’ experiences. The third author read a selection of transcripts and participated in discussions throughout the analytical process. The first and second authors conducted the initial coding, identifying meaningful data relevant to the study aim. All three authors subsequently engaged in reflective discussions to examine, compare, and interpret the codes, which were then organised into potential themes and subthemes. The themes were reviewed and refined to ensure coherence with both the coded extracts and the quotes from the transcripts. Through an iterative process of dialogue, critical reflection, and repeated engagement with the data, the authors collaboratively refined, defined, and named the themes. This process facilitated research triangulation and contributed to a nuanced interpretation of the findings. The analysis ultimately resulted in three themes that addressed the study aim. Examples of the analytical process are provided in Table 1.

**Table 1:** Examples from the analysis process

Quotes/meaning-bearing units	Codes	Subthemes	Themes
*Behaviour*,* such as a lot of restlessness*,* more than usual*,* can be a sign of pain. Patients with dementia are often restless.*	How nurses use observation during pain assessment	Assessment procedures	Pain assessment is similar to detective work
*We were two very familiar people at work*,* so we very much agreed that here there was something different than what it usually is.*	The advantage of full-time employees/ known personnel	Knowledge of the patient	
*So*,* I’m very interested in listening to my colleagues and what they themselves observe*,* so I can observe together with them and make an assessment together with them.*	Collaboration with colleagues	Use of collaboration in pain assessment	Pain assessment of patients with dementia requires collaboration
*And we often see that they are very quick to ask for calm instead of trying to find out what is really behind the unrest.*	Challenges surrounding unskilled workers	Challenges with pain assessment	Nurses’ desire to improve the quality of pain assessment for patients with dementia
*That mapping tool is really important to reach the goal.*	Benefits of mapping tools	Thoughts on improvement work	

### Reflexivity

All three researchers are registered nurses with professional experience in nursing homes and caring for people living with dementia. This background provided important contextual knowledge and enabled both the first- and second authors to ask relevant follow-up questions during the interviews. Consistent with reflexive thematic analysis [32], the authors recognised that their professional experiences and preunderstandings could shape data collection, interpretation, and theme development. Reflexivity was therefore maintained throughout the study through reflective notes and continuous discussions of assumptions, emerging interpretations, and analytic decisions among all the authors. These discussions helped challenge individual perspectives and supported a reflexive and interpretive analytic process, acknowledging the researchers’ active role in the construction of themes.

### Ethical considerations

Ethical considerations were carefully addressed throughout the study. The project was registered with the Norwegian Agency for Shared Services in Education and Research (SIKT; project number 745436) and was undertaken in accordance with the ethical principles outlined in the Declaration of Helsinki [33]. Before participation, participants were provided with comprehensive written and verbal information regarding the study aims, procedures and confidentiality arrangements. They also received written and oral information that their participation in the study was voluntary and that they could withdraw their consent at any time. Written informed consent was obtained from all participants before data collection began. To safeguard confidentiality, the interview data were anonymised and stored electronically on password-protected computers secured by two-factor authentication. Access to the data was limited to the authors involved in the study. The participants did not receive any financial remuneration or other incentives for their participation.

## Results

Eight nurses working with patients with dementia in two nursing homes from different municipalities in western Norway participated in this study. All participants were female, with nursing experience ranging from 1.5 to 33 years, and all had experience caring for individuals with dementia. When analysing the empirical data, three main themes were identified: (1) pain assessment is similar to detective work; (2) pain assessment of patients with dementia requires collaboration; and (3) nurses’ desire to improve the quality of pain assessment for patients with dementia.

### Pain assessment is similar to detective work

All the nurses reported that NPS, such as restlessness, aggression, agitation, and other behavioural changes, were important behavioural indicators to consider during pain assessment. As patients with dementia may have difficulty expressing pain verbally, behavioural changes are an observation that nurses must be aware of. These expressions are often interpreted as possible manifestations of pain, particularly if patients are unable to communicate their discomfort. Several nurses mentioned that they use different methods of observing and assessing whether patients with dementia could experience pain, trying to obtain a complete overview by ruling out other possible causes of patient discomfort. One of the nurses emphasised her way of looking at the bigger picture like being a detective, as there is no single clear method to assess pain in patients with dementia: ‘*It is about searching and trial and error*,* and it’s kind of like detective work*,* but the path to get there can be very difficult’* (Nurse 1).

Several nurses reported frequently using different assessment tools, particularly the MOBID-2 pain scale and a 24-h behaviour registration form to track changes in behaviour. In the latter, colour codes are applied throughout the day, each corresponding to specific types of patient behaviour potentially related to pain. According to some participants, behavioural indicators include NPS such as restlessness, aggression, and agitation, as well as changes in sleep patterns and appetite, as one participant explained:

*We use a lot of mapping forms with colouring to see if there is anything specific during the day or if there is something beforehand that could cause it. We try to look for a pattern [in behaviour]* (Nurse 5).

When nurses were unable to identify a specific cause of pain through assessment, they described relying on a range of nonpharmacological interventions, including massage, calming music, hot water bottles, communication, and physical closeness, after first addressing the patient’s basic needs.

Rather than focusing solely on identifying and treating pain, nurses sought to alleviate distress and enhance comfort while continuing to observe the patient’s response. Some nurses described pain assessment as resembling detective work, involving a process of elimination in which analgesics were sometimes administered to explore whether pain might be an underlying cause of behavioural changes, such as agitation and restlessness, in patients with dementia:

*Testing Paracetamol to see if it works is done to assess whether the patient is actually experiencing pain* (Nurse 2).

### Pain assessment of patients with dementia requires collaboration

The nurses emphasised that developing a comprehensive understanding of each individual patient was a fundamental aspect of pain assessment in dementia care. Such knowledge involved gathering information about the patient’s life history, habitual behaviours, preferences, and needs prior to the onset of dementia, as well as consulting medical records and previous assessments. Knowledge of the patient’s previous and present life, together with observations of behavioural indicators, enabled nurses to identify deviations from the patient’s usual patterns of behaviour and functioning, which were often regarded as potential indicators of pain. The nurses emphasised the importance of informing and involving relatives in the pain assessment process when working with patients with dementia. The relatives often have valuable knowledge of the patient’s life history and can provide valuable insights into the patient’s behavioural patterns. In this context, relatives’ insights were perceived as valuable in supporting nurses’ efforts to differentiate between psychological distress and physical pain. One of the participants provided examples of the questions she asked relatives, such as: *How was the patient before he got dementia? How has the patient expressed pain [before]? (Nurse 7).*

Some nurses highlighted that patients with dementia should not automatically be assumed to be unable to report pain. When possible, the patient’s own expressions and descriptions of pain were regarded as important sources of information in the assessment process. One nurse emphasised that engaging the patient in pain assessment was just as important as collaborating with relatives and other healthcare professionals: *As long as they are able to communicate their pain. We must not forget that many are still capable of doing so. They can express their needs*,* even with a dementia diagnosis (Nurse 8).*

All nurses in the study described interprofessional collaboration with doctors, physiotherapists, psychiatrists, dental hygienists, social educators, and other healthcare workers as essential when assessing pain in patients with dementia in nursing homes.

The nurses described using various tools to facilitate interprofessional collaboration. Some nurses referred to structured approaches, including TIME, as a framework for interdisciplinary discussion. They considered TIME-meetings particularly valuable for assessing newly developed behaviours in patients, and discussing possible underlying causes of behaviour changes, such as pain.

Several nurses reported that pain-related concerns were also discussed in VIPS framework meetings, where the patient’s individual and daily needs, experiences, and perspectives were central to the assessment process. The nurses explained that these interprofessional face-to-face meetings facilitated collaborative reflection and supported the identification of personalized approaches to pain assessment and care:

*We have started something called VIPS meetings. There*,* we discuss one patient at a time. We identify the issue*,* map it out for the next meeting*,* and then everyone goes through it together*,* and we make out a plan for further interventions* (Nurse 5).

Collaboration with colleagues in pain assessment for patients with dementia was described as a crucial part of the evaluation. Gathering information, observing, and assessing together with colleagues over time was something that almost all the participants highlighted as useful. Some of the nurses also work in mobile nursing teams, which means that they are not tied to just one nursing home, and are entirely dependent on collaborating with other healthcare professionals for continuous observation:

*We are often dependent on others making the assessments for us*,* such as healthcare workers and assistants* (Nurse 8).

### Nurses’ desire to improve the quality of pain assessment for patients with dementia

All the nurses expressed a desire to improve the quality of pain assessment for patients with dementia. Their reflections revealed a need for greater confidence and competence in assessing pain in patients with limited verbal communication. Several nurses described uncertainty when interpreting body language and behavioural changes and questioned whether the observed expressions reflected physical pain, psychological distress, or other unmet needs. Due to these challenges, nurses reported rarely using numerical or subjective pain scales. They highlighted the difficulty of distinguishing between physical and psychological pain in patients with absent language, making the assessment highly complex, as one participant said:

*It is complicated to assess a dementia patient’s pain because pain is something subjective. It is only the person who has pain who feels it*,* and when you cannot properly respond when you try to communicate and ask*,* it becomes a problem * (Nurse 8).

Most nurses emphasised that the nursing homes where they work focus on assessing pain in patients with dementia, but that the quality of care could be improved if various challenges were addressed. Some nurses also expressed a desire to make greater use of nonpharmacological interventions during pain assessment and care. Most of the nurses reported that assessment tools are used infrequently and argued that they should be applied more consistently, particularly in settings where nurses are regularly present. At the same time, several nurses highlighted the need for practice and training in the use of these tools, as they felt that they were often not used correctly. One participant explained:

*We struggle with the fact that suddenly there are two days which the assessment tools are not filled out*,* or they have not filled out at night. There is a significant gap in what people are capable of. It becomes like a puzzle*,* requiring interpretation of the results* (Nurse 7).

The nurses suggested that greater knowledge of dementia care could improve the quality of pain assessment and reduce unnecessary use of analgesics. They described pain assessment as a time-consuming process that requires continuity of care, familiarity with the individual patient, and competent staff. However, staff turnover, sickness absence, time pressures, and the use of unskilled personnel often limit opportunities for thorough assessment. Several nurses described how these constraints created uncertainty when interpreting behavioural changes and increased the risk that distress could be misinterpreted. One nurse described the situation as follows: *There is a lot that can be initiated*,* but unfortunately*,* we have too few resources. Many medications could have been used less if we only had enough staff* (Nurse 6).

Several nurses highlighted that familiarity and competence are closely connected. Nurses who worked regularly with the same patients reported being better able to identify subtle behavioural changes that might indicate pain, as one participant said:

*Familiarity and qualification are the key elements when working with people with dementia (…) I work full time and see them every day*,* and after a while*,* it becomes very easy to observe any variations that may indicate pain *(Nurse 1).

Moreover, some nurses identified management as a key factor influencing pain assessment. One nurse emphasised the need for training and education in various procedures, suggesting that management should play an active role in initiating and supporting a focus on pain assessment, as one participant stated:

*You must have support from the management. You need the management to be aware of it and facilitate it*,* and for the management to try to get a sense of what is happening in the department. I feel that there is a very significant difference in this* (Nurse 7).

## Discussion

In this study, the aim was to explore nurses’ experiences of assessing pain in patients with dementia in nursing homes. Although self-report is widely regarded as the standard for subjective pain assessment [34] this approach can be particularly challenging in nursing home residents with dementia because cognitive impairment often affects communication, self-expression, and abstract thinking. As dementia progresses, patients may experience increasing difficulty in both answering questions about pain and verbally conveying their pain experiences [35]. Therefore, healthcare professionals such as nurses are required to rely on alternative sources of information during the assessment process.

This study provides detailed insight into the practical challenges faced by nurses, emphasising the need for structured training, interprofessional collaboration, and organizational support to improve pain assessment and management for this underserved patient population. Similar to results from a systematic review [36] our findings revealed that in-depth knowledge of the patient, the use of various tools for pain assessment, and effective collaboration are key factors in the assessment of pain in this patient group.

All the nurses who participated in the study reported that pain assessment in patients with dementia is challenging. Effective pain assessment requires both competence in dementia care and strong clinical assessment skills. Several nurses compared it, in various ways, to detective work, requiring careful observation to identify the cause of discomfort. The nurses’ description of pain assessment as “detective work” can also partly reflect the challenge of differentiating pain from behavioural and psychological symptoms of dementia. This metaphor is consistent with recent evidence describing pain assessment in dementia as a process characterised by uncertainty and clinical judgement, in which healthcare professionals must differentiate pain from behavioural and psychological symptoms of dementia that often present with overlapping manifestations [37].

Moreover, previous research has shown that pain is associated with increased agitation and behavioural disturbances in nursing home residents with dementia and may consequently be hidden behind symptoms that are often attributed to the dementia itself [35, 38]. Knowledge about the patient will therefore significantly influence this ‘detective work’, together with an understanding of dementia and its impact on the patient is also essential for performing effective pain assessments [35, 39].

The nurses’ descriptions suggest that pain assessment in people with dementia extends beyond the identification of isolated symptoms. Instead, assessment appears to involve an ongoing interpretive process, in which observations are continuously evaluated against competing explanations [40]. Changes in behaviour, facial expressions, withdrawal, agitation, or resistance to care can indicate pain, but can also reflect NPS, unmet needs, environmental stressors, or illness-related factors. The findings therefore highlight pain assessment as a process of clinical reasoning characterised by uncertainty and the continuous interpretation of often ambiguous information. According to the Dementia Care Plan, individuals with dementia who exhibit behavioural or psychological symptoms should be assessed for pain as part of nursing care [41].

In this study, the nurses stated that pain assessment is particularly challenging when patients have limited verbal communication and that there is no standardised procedure for evaluating pain. They emphasised the importance of considering all aspects of the patient, including conducting a systematic examination from head to toe to detect signs of pain and employing TIME [19] during VIPS meetings [20] to differentiate these from behavioural changes caused by dementia. However, the nurses’ descriptions suggest that pain assessment is not simply a matter of identifying symptoms but a process of generating and evaluating competing explanations for behavioural changes. In this sense, pain assessment may be understood as a process of clinical reasoning in which nurses continuously formulate, test, and revise hypotheses about possible sources of distress [40].

Similar to results from another study [42], collaboration with relatives was also described as valuable, providing second-hand knowledge of the patients’ pain-related behaviours prior to the onset of dementia. By providing knowledge of the person’s usual behaviour and previous responses to pain, relatives may assist nurses in distinguishing pain-related distress from other possible causes of behavioural change. This can be particularly important when pain assessment relies on observation rather than self-report [43].

The nurses reported that familiarity with patients over time makes it easier to recognise signs of pain through body language, behaviour, and facial expressions. Together, information from relatives [43] and nurses’ familiarity with the patients [35] appeared to provide an important foundation for recognising pain-related changes. Because behavioural expressions of pain are often subtle and highly individual, nurses rely on knowledge of the person’s habitual behaviour to recognise meaningful changes. Rather than interpreting behaviours in isolation, nurses compare current observations with their understanding of the patient’s usual patterns of communication, activity, mood, and social interaction. Full-time employment enhances continuity and facilitates the detection of changes in patients [16]. Management that promotes a full-time culture contributes to greater continuity of patient care, predictability, and workplace stability, benefiting both patients and staff [44]. However, continuity alone may not be sufficient. The ability to detect and correctly interpret pain-related changes requires clinical knowledge, experience, and competence in dementia care. Familiarity with the patient and nursing expertise therefore appear to be important prerequisites for effective pain assessment in this population [35, 43].

The nurses explained that assessing pain in patients with dementia also relies heavily on teamwork and collaboration with other healthcare professionals. Interprofessional collaboration is also essential for developing comprehensive, individual care plans [45]. To meet these requirements, many Norwegian municipalities have implemented models for person-centred care. Interprofessional collaboration is recognised as critical for enhancing overall competence in health and care services [41].

Nurses’ positive experiences with the VIPS framework [20] and the TIME model [19] could indicate that structured collaborative frameworks help manage the uncertainty inherent in pain assessment among people with dementia. Rather than replacing clinical judgment, these models appear to support clinical reasoning through systematic reflection, the integration of different perspectives, and shared decision-making among healthcare professionals. The VIPS framework emphasises understanding behavioural changes within the context of an individual’s health status, personal history, preferences, and social environment and has been associated with improved person-centred care and collaboration among staff [46–47]. Similarly, the TIME model promotes systematic assessment and interdisciplinary reflection to support the interpretation of complex behavioural symptoms [48] and has been linked to reduced NPS and improved quality of life among people with dementia [49–50]. This perspective aligns closely with the nurses’ descriptions of pain assessment as a process of exploring multiple possible explanations for behavioural change. Structured frameworks such as VIPS and the TIME model could therefore support clinical reasoning by encouraging healthcare professionals to interpret behavioural expressions in relation to the individual’s broader context rather than as isolated symptoms. The findings of this study suggest that these frameworks could support more person-centred and individualised assessment and care. Despite this, several Norwegian municipalities have yet to implement these person-centred care models [40]. To enhance nurses’ competence in pain assessment, effective routines and training in these models are essential.

The nurses indicated that assessment tools are effective for evaluating pain in patients with dementia. Proper use of these tools simplifies and clarifies the assessment process. However, they expressed concern that tools are underused. Guidelines recommend using mapping tools, particularly for patients with communication difficulties. The MOBID-2 pain scale is recognised as an effective method for assessing whether patients are experiencing physical pain [3]. Evidence suggests that the use of the MOBID-2 enables timely identification and management of pain, which may facilitate earlier pain relief and reduce NPS [23, 51–52].

Interestingly, evidence suggests that systematic assessment alone may not be sufficient to improve pain outcomes. Rostad et al. [53] reported that the regular use of a standardized pain assessment tool did not lead to significant reductions in pain scores or changes in analgesic use among nursing home residents with severe dementia, despite repeated assessments over a 12-week period. Although assessment tools could support observation and documentation, uncertainty remains because assessment findings must still be interpreted and translated into clinical action. Pain assessment in dementia care may therefore require not only systematic observation, but also clinical reasoning, professional judgment, and knowledge of the individual patient [53].

The nurses in this study reported that limited training and the absence of structured routines for the use of assessment tools contributed to their underutilisation. This might increase the risk of uncertainty and inconsistency in pain assessment among patients with dementia. Previous research has suggested that successful implementation of pain assessment tools depends not only on the availability of the tools themselves but also on organisational support, staff training, and opportunities to develop competence through practice [54–55]. Furthermore, education in the use of pain assessment tools has been associated with improved assessment efficiency, better integration of pain assessment into daily clinical practice, and more timely pain management [56]. These findings suggest that assessment tools alone are insufficient; their effectiveness depends on nurses having the knowledge, confidence, and organisational support necessary to incorporate them into clinical reasoning and everyday practice.

Management should establish routines and provide training to ensure effective use, acknowledging that, by law, Norwegian nurses bear independent responsibility for performing their duties competently [57].

In this study, nurses employed nonpharmacological interventions, such as massage, close human contact [58], music, and communication [59], to manage pain in patients with dementia. Previous research has indicated that such interventions may help relieve both pain and NPS through supportive interpersonal interactions and sensory stimulation [58–59]. Rather than addressing a single dimension of suffering, nonpharmacological interventions could simultaneously support physical, psychological, social, and emotional well-being.

The incorporation of nonpharmacological measures helps nurses differentiate between physical and psychological pain, which is often challenging [60]. Similarly, the nurse participants in the current study also suggested that patients’ responses to these interventions may provide valuable information during the assessment process, thereby contributing to a broader understanding of pain-related distress and supporting more individualised care. However, similar to results from two systematic reviews [16–17] our findings suggest that limited time, high workloads, and staffing challenges may constrain nurses’ ability to conduct comprehensive pain assessments in people with dementia.

Because pain assessment in this patient group often relies on observation, interpretation, and follow-up over time, insufficient staffing and fragmented continuity can reduce opportunities for thorough assessment and increase the risk that pain-related symptoms are overlooked. The nurses described how high workloads, part-time staffing, limited professional focus, and reliance on unskilled personnel to cover vacant shifts affected the quality of care and reduced the time available for pain assessment, a finding consistent with previous research [61]. Efforts to promote continuity through a full-time work culture might therefore not only improve staffing stability but also strengthen opportunities for professional development, learning, and the recruitment of skilled personnel [62–63].

### Strengths and limitations

This study has several strengths and limitations. One of the strengths is that using individual interviews as a qualitative research method enabled a more in-depth exploration of the study’s aim. The nurses who participated were employed at two different nursing homes. The inclusion of nurses from two distinct nursing homes enhanced the data’s contextual variety, allowing for a broader exploration of pain assessment practices across different institutional settings. Conducting only eight interviews may be seen as a limitation. However, the participants were experienced nurses, hence ensuring the collection of rich qualitative data and providing a comprehensive perspective on the topic. The interviews were carried out individually, thereby minimising the influence of external factors on participants’ responses, which is considered advantageous.

The findings should be considered as one of the methodological limitations. Six interviews were conducted at one nursing home, whereas only two were conducted at the other. Consequently, the findings might be more strongly influenced by the organisational culture, routines, and experiences of nurses from the nursing home that contributed most participants. Although similar topics emerged in both settings, the unequal distribution of participants may have limited the range of the perspectives represented in the data and introduced uncertainty about the transferability of the findings to other nursing home contexts.

Another limitation of this study is that all the participants were women. While this reflects the predominance of women in the nursing profession and recruitment context, the absence of male participants could have limited the diversity of perspectives represented in the data. However, given that the study aimed to explore nurses’ experiences of pain assessment in patients with dementia rather than to compare experiences across genders, the findings are considered relevant to the study aim.

### Implications for clinical practice

The findings indicate the importance of supporting nurses in conducting comprehensive, person-centred pain assessments for people living with dementia. This requires adequate staffing, sufficient time for systematic observation rather than just “detective work”, access to validated pain assessment tools such as CNPI, MOBID-2, and Doloplus-2, and opportunities for interdisciplinary collaboration. Strengthening nurses’ competence in interpreting behavioural expressions of pain and integrating pharmacological and non-pharmacological approaches may facilitate earlier recognition and management of pain, ultimately improving comfort, reducing NPS, and enhancing the quality of dementia care.

## Conclusion

The findings of this study suggest that nurses experience pain assessment in patients with dementia in nursing homes as a complex and challenging process. Limited verbal communication, behavioural and psychological symptoms, and uncertainty regarding the underlying causes of distress require nurses to rely on observation, clinical judgment, and knowledge of the individual patient. The nurses described pain assessment as a process of exploring multiple possible explanations for behavioural change rather than identifying a single, clear indicator of pain.

Familiarity with the patient, collaboration with relatives and other healthcare professionals, and the use of assessment tools were perceived as important sources of information in the assessment process. At the same time, the findings indicate that competence, continuity, and opportunities for systematic reflection may influence nurses’ ability to recognise and interpret pain-related expressions in people with dementia.

This study provides insight into nurses’ experiences of pain assessment in dementia care and highlights the complexity of clinical decision-making when pain cannot be assessed through self-report. Further research is needed to explore how nurses can be supported in conducting pain assessments in people with dementia across different care settings.

## Supplementary Information

Below is the link to the electronic supplementary material.

Supplementary Material 1

## Data Availability

The data sets used and analysed in this study are unavailable due to the protection of the study participants’ privacy following the project approval by the Norwegian Agency for Shared Services in Education and Research, project number: 745436.

## References

[1] Alzheimer's Disease International. World Alzheimer Report 2024 [Internet]. London: Alzheimer's Disease International; 2024 Sep 24 [cited 2025 Feb 6]. Available from: https://www.alzint.org/resource/world-alzheimer-report-2024/.

[2] Norwegian Institute of Public Health. Dementia [Internet]. Oslo: Norwegian Institute of Public Health; 2026 [cited 2026 Jul 6]. Available from: https://www.fhi.no/he/fr/folkehelserapporten/ikke-smittsomme/demens/.

[3] Norwegian Directorate of Health. Guidelines on dementia [Internet]. Oslo: Norwegian Directorate of Health; 2017 Aug 16 [updated 2024 Feb 23; cited 2025 Feb 7]. Available from: https://www.helsedirektoratet.no/retningslinjer/demens/om-demens.

[4] Volkmer A, Cross L, Highton L, Jackson C, Smith C, Brotherhood E, et al. Communication is difficult’: Speech, language and communication needs of people with young onset or rarer forms of non-language led dementia. Int J Lang Commun Disord. 2024;59(4):1553–77. 10.1111/1460-6984.13018. 38329409

[5] Warren A. Behavioral and psychological symptoms of dementia as a means of communication: considerations for reducing stigma and promoting person-centered care. Front Psychol. 2022;29(13):875246. 10.3389/fpsyg.2022.875246. PMC9002111 35422728

[6] Cole CS, Blackburn J, Carpenter JS, Chen CX, Hickman SE. Pain and associated factors in nursing home residents. Pain Manag Nurs. 2023;24(4):384–92. 10.1016/j.pmn.2023.03.002. PMC10440293 37003932

[7] Helvik AS, Bergh S, Šaltytė Benth J, Selbaek G, Husebo BS, Tevik K. Pain in nursing home residents with dementia and its association to quality of life. Aging Ment Health. 2022;26(9):1787–97. 10.1080/13607863.2021.1947968. 34251936

[8] Helvik AS, Bergh S, Tevik K. A systematic review of prevalence of pain in nursing home residents with dementia. BMC Geriatr. 2023;23(1):641. 10.1186/s12877-023-04340-z. PMC10566134 37817061

[9] Cohen SP, Vase L, Hooten WM. Chronic pain: an update on burden, best practices, and new advances. Lancet. 2021;397(10289):2082–97. 10.1016/S0140-6736(21)00393-7. 34062143

[10] Husak AJ, Bair MJ. Chronic Pain and Sleep Disturbances: A Pragmatic Review of Their Relationships, Comorbidities, and T reatments. Pain Med. 2020;21(6):1142–52. 10.1093/pm/pnz343. 31909797

[11] LaRowe LR, Miaskowski C, Miller A, Mayfield A, Keefe FJ, Smith AK, et al. Chronic Pain and Pain Management in Older Adults: Protocol and Pilot Results. Nurs Res. 2024;73(1):81–8. 10.1097/NNR.0000000000000683. PMC10829063 37582291

[12] Mullins S, Hosseini F, Gibson W, Thake M. Physiological changes from ageing regarding pain perception and its impact on pain management for older adults. Clin Med. 2022;22(4):307–10. 10.7861/clinmed.22.4.phys. PMC9345212 35882493

[13] Bell T, Franz CE, Kremen WS. Persistence of pain and cognitive impairment in older adults. J Am Geriatr Soc. 2022;70(2):449–58. 10.1111/jgs.17542. PMC8821128 34741304

[14] Chen J, Wang X, Xu Z. The relationship between chronic pain and cognitive impairment in the elderly: a review of current evidence. J Pain Res. 2023;16:2309–19. 10.2147/JPR.S416253. PMC10335316 37441267

[15] Cravello L, Di Santo S, Varrassi G, Benincasa D, Marchettini P, de Tommaso M, et al. Chronic pain in the elderly with cognitive decline: a narrative review. Pain Ther. 2019;8(1):53–65. 10.1007/s40122-019-0111-7. PMC6513941 30666612

[16] Jonsdottir T, Gunnarsson EC. Understanding nurses’ knowledge and attitudes toward pain assessment in dementia: a literature review. Pain Manag Nurs. 2021;22(3):281–92. 10.1016/j.pmn.2020. 33334680

[17] Liao YJ, Jao YL, Berish D, Hin AS, Wangi K, Kitko L, Mogle J, Boltz M. A systematic review of barriers and facilitators of pain management in persons with dementia. J Pain. 2023;24(5):730–41. 10.1016/j.jpain.2022.12.014. 36634886

[18] Tsai YIP, Browne G, Inder KJ. Documented nursing practices of pain assessment and management when communicating about pain in dementia care. J Adv Nurs. 2022;78(10):3174–86. 10.1111/jan.15251. PMC9545301 35436007

[19] Lichtwarck B, Selbæk G, Kirkevold Ø, Rokstad AMM, Saltyte JB, Bergh S. TIME – Targeted interdisciplinary model for evaluation and treatment of neuropsychiatric symptoms: protocol for an effectiveness-implementation cluster randomized hybrid trial. BMC Psychiatry. 2016;16:233. 10.1186/s12888-016-0944-0. PMC4942955 27406242

[20] Røsvik J, Brooker D, Mjørud M, Kirkevold Ø. What is person-centred care in dementia? Clinical reviews into practice: The development of the VIPS practice model. Reviews Clin Gerontol. 2013;23(2):155–63. 10.1017/S0959259813000014.

[21] Hølen J, Saltvedt I, Fayers P, Hjermstad M, Loge J, Kaasa S. Doloplus-2, a valid tool for behavioural pain assessment? BMC Geriatr. 2007;7(1):29. 10.1186/1471-2318-7-29. PMC2234400 18093294

[22] Hølen JC, Saltvedt I, Fayers PM, Bjørnnes M, Stenseth G, Hval B, Filbet M, Loge JH, Kaasa S. The Norwegian Doloplus-2, a tool for behavioural pain assessment: translation and pilot-validation in nursing home patients with cognitive impairment. Palliat Med. 2005;19(5):411–7. 10.1191/0269216305pm1031oa. 16111065

[23] Husebø BS, Strand LI, Moe-Nilssen R, Husebø SB, Ljunggren AE. Pain in older persons with severe dementia. Psychometric properties of the Mobilization–Observation–Behaviour–Intensity–Dementia (MOBID-2) Pain Scale in a clinical setting. Scan J Caring Sci. 2010;24(2):380–91. 10.1111/j.1471-6712.2009.00710.x. 20210897

[24] Nygaard HA, Jarland M. The Checklist of Nonverbal Pain Indicators (CNPI): testing of reliability and validity in Norwegian nursing homes. Age Ageing. 2006;35(1):79–81. 10.1093/ageing/afj008. 16364939

[25] El-Tallawy SN, Ahmed RS, Shabi SM, Al-Zabidi FZ, Zaidi ARZ, Varrassi G, et al. The challenges of pain assessment in geriatric patients with dementia: a review. Cureus. 2023;15(11):e49639. 10.7759/cureus.49639. PMC10755634 38161929

[26] Pu L, Barton M, Kodagoda MWG, Okada M, Todorovic M, Moyle W. Pain Assessment and Management in Dementia Care: Qualitative Perspectives of People with Dementia, Their Families, and Healthcare Professionals. J Clin Nurs. 2025;34(7):2933–47. 10.1111/jocn.17771. PMC12181161 40200556

[27] Spichiger F, Volken T, Bosshard G, Zigan N, Blanc G, Büscher A, et al. Pain Interventions for people with dementia: a quasi-experimental study. BMC Palliat Care. 2022;21(1):228. 10.1186/s12904-022-01118-9. PMC9798691 36581883

[28] Kodagoda MWG, Pu L, Todorovic M, Moyle W. Nurses’ Beliefs About Pain Assessment in Dementia: A Qualitative Study Informed by the Theory of Planned Behaviour. J Clin Nurs. 2024;33(12):4795–808. 10.1111/jocn.17417. PMC11579553 39209772

[29] Polit DF, Beck CT. Essentials of nursing research: appraising evidence for nursing practice. 10th ed. Philadelphia (PA): Wolters Kluwer; 2021. 448.

[30] Hacking I. The social construction of what? Cambridge (MA): Harvard University Press; 1999. p. 261.

[31] Malterud K, Siersma VD, Guassora AD. Sample Size in Qualitative Interview Studies: Guided by Information Power. Qual Health Res. 2016;26(13):1753–60. 10.1177/1049732315617444. 26613970

[32] Braun V, Clarke V. Reflecting on reflexive thematic analysis. Qual Res Sport Exerc Health. 2019;11(4):589–97. 10.1080/2159676X.2019.1628806.

[33] World Medical Association. World Medical Association Declaration of Helsinki: ethical principles for medical research involving human participants [Internet]. Ferney-Voltaire (France): World Medical Association; 2024 [cited 2025 Oct 1]. Available from: https://www.wma.net/what-we-do/medical-ethics/declaration-of-helsinki/. 10.1001/jama.2024.21972 39425955

[34] Jensen MP, Miró J, Euasobhon P. Assessing pain intensity: critical questions for researchers and clinicians. Anaesthesia. 2024;79(2):114–8. 10.1111/anae.16150. PMC10841457 38058201

[35] McAuliffe L, Nay R, O’Donnell M, Fetherstonhaugh D. Pain assessment in older people with dementia: literature review. J Adv Nurs. 2009;65(1):2–10. 10.1111/j.1365-2648.2008.04861.x. 19016920

[36] Pu L, Gamage MK, Li N, Barton M, Feenstra M, Todorovic M, Moyle W. Reviewing the landscape of the decision-making process for pain assessment and management for people living with dementia: A systematic review. Geriatr Nurs. 2025;61:50–63. 10.1016/j.gerinurse.2024.10.047. 39541632

[37] Lichtner V, Dowding D, Esterhuizen P, Closs SJ, Long AF, Corbett A, Briggs M. Pain assessment for people with dementia: a systematic review of systematic reviews of pain assessment tools. BMC Geriatr. 2014;14:138. 10.1186/1471-2318-14-138. PMC4289543 25519741

[38] Nowak T, Neumann-Podczaska A, Deskur-Śmielecka E, Styszyński A, Wieczorowska-Tobis K. Pain as a challenge in nursing home residents with behavioral and psychological symptoms of dementia. Clin Interv Aging. 2018;13:1045–51. 10.2147/CIA.S157246. PMC5973322 29872283

[39] Achterberg W, Lautenbacher S, Husebo B, Erdal A, Herr K. Pain in dementia. Pain Rep. 2019;5(1):e803. 10.1097/PR9.0000000000000803. PMC7004504 32072098

[40] Overen CK, Larsson M, Hillestad AH, Karlsson I, Eriksen S. The process of pain assessment in people with dementia living in nursing homes: a scoping review. Palliat Care Soc Pract. 2025;19:1–19. 10.1177/26323524241308589. PMC11705334 39776523

[41] Norwegian Government. Dementia Plan 2025 [Internet]. Oslo: Norwegian Government; 2022 [cited 2025 Oct 1]. Available from: https://www.regjeringen.no/en/dokumenter/dementia-plan-2025/id2788070/.

[42] Nguyen H, Eccleston CE, Doherty KV, Jang S, McInerney F. Communication in dementia care: Experiences and needs of carers. Dementia. 2022;21(4):1381–98. 10.1177/14713012221080003. 35333128

[43] Pu L, Chen H, Jones C, Moyle W. Family involvement in pain management for people living with dementia: an integrative review. J Fam Nurs. 2023;29(1):43–58. 10.1177/10748407221114502. 35898190

[44] Magerøy MR, Wiig S. The effect of full-time culture on quality and safety of care–a literature review. Int J Health Gov. 2023;28(1):68–81. 10.1108/IJHG-11-2022-0106.

[45] The Health and Care Services Act. Act of 24 June 2011 No. 30 relating to municipal health and care services, etc. Oslo: Ministry of Health and Care Services; 2011. https://lovdata.no/dokument/NL/lov/2011-06-24-30.

[46] Mjørud M, Røsvik J. You can tell it works–experiences from using the VIPS practice model in primary healthcare. Dementia. 2022;21(2):579–94. 10.1177/14713012211053979. 34854785

[47] Røsvik J, Mjørud M. We must have a new VIPS meeting soon!’ Barriers and facilitators for implementing the VIPS practice model in primary health care. Dementia. 2021;20(8):2649–67. 10.1177/14713012211007409. PMC8670746 33870756

[48] Hoel KA, Lichtwarck B, Væringstad A, Feiring IH, Rokstad AMM, Selbæk G, et al. Correction: Targeted interdisciplinary model for evaluation and treatment of neuropsychiatric symptoms (TIME) in home care services: a cluster randomized feasibility trial. BMC Health Serv Res. 2022;22(1):915. 10.1186/s12913-022-08308-4. PMC9284742 35836167

[49] Lichtwarck B, Selbaek G, Kirkevold Ø, Rokstad AMM, Benth JŠ, Lindstrøm JC, et al. Targeted interdisciplinary model for evaluation and treatment of neuropsychiatric symptoms: a cluster randomized controlled trial. Am J Geriatr Psychiatry. 2018;26(1):25–38. 10.1016/j.jagp.2017.05.015. 28669575

[50] Lichtwarck B, Myhre J, Goyal AR, Rokstad AMM, Selbaek G, Kirkevold Ø, Bergh S. Experiences of nursing home staff using the targeted interdisciplinary model for evaluation and treatment of neuropsychiatric symptoms (TIME) – a qualitative study. Aging Ment Health. 2019;23(8):966–75. 10.1080/13607863.2018.1464116. 29669442

[51] Husebo B, Ostelo R, Strand LI. The MOBID-2 pain scale: Reliability and responsiveness to pain in patients with dementia. Eur J Pain. 2014;18(10):1419–30. 10.1002/ejp.507. PMC4230478 24799157

[52] Smith TO, Harvey K. Psychometric properties of pain measurements for people living with dementia: a COSMIN systematic review. Eur Geriatr Med. 2022;13(5):1029–45. 10.1007/s41999-022-00655-z. PMC9553783 35622210

[53] Rostad HM, Utne I, Grov EK, Småstuen MC, Puts M, Halvorsrud L. The impact of a pain assessment intervention on pain score and analgesic use in older nursing home residents with severe dementia: a cluster randomised controlled trial. Int J Nurs Stud. 2018;84:52–60. 10.1016/j.ijnurstu.2018.04.017. 29763832

[54] Felton N, Lewis JS, Cockburn SJ, Hodgson M, Dawson S. Pain assessment for individuals with advanced dementia in care homes: a systematic review. Geriatrics. 2021;6(4):101. 10.3390/geriatrics6040101. PMC8544573 34698157

[55] Norwegian Labour Inspection Authority. Section 8-1. General requirements for training [Internet]. 2025 [cited 2025 Feb 17]. Available from: https://www.arbeidstilsynet.no/en/laws-and-regulations/regulations/regulations-concerning-organisation-management-and-employee-participation/8/section8-1/.

[56] Tsai YIP, Browne G, Inder KJ. The effectiveness of interventions to improve pain assessment and management in people living with dementia: A systematic review and meta-analyses. J Adv Nurs. 2021;77(3):1127–40. 10.1111/jan.14660. 33222273

[57] The Health Personnel Act. Act of 2 July 1999 No. 64 relating to health personnel, etc. Oslo: Ministry of Health and Care Services; 1999. https://lovdata.no/dokument/NL/lov/1999-07-02-64.

[58] Anderson AR, Deng J, Anthony RS, Atalla SA, Monroe TB. Using complementary and alternative medicine to treat pain and agitation in dementia: a review of randomized controlled trials from long-term care with potential use in critical care. Crit Care Nurs Clin North Am. 2017;29(4):519–37. 10.1016/j.cnc.2017.08.010. PMC5687304 29107312

[59] Lam HL, Li WTV, Laher I, Wong RY. Effects of music therapy on patients with dementia—A systematic review. Geriatrics. 2020;5(4):62. 10.3390/geriatrics5040062. PMC7709645 32992767

[60] Liao YJ, Parajuli J, Jao YL, Kitko L, Berish D. Non-pharmacological interventions for pain in people with dementia: A systematic review. Int J Nurs Stud. 2021;124:104082. 10.1016/j.ijnurstu.2021.104082. 34607070

[61] Kang Y, Hur Y. Nurses’ experience of nursing workload-related issues during caring patients with dementia: a qualitative meta-synthesis. Int J Environ Res Public Health. 2021;18(19):10448. 10.3390/ijerph181910448. PMC8508301 34639748

[62] Johannessen T, Ree E, Aase I, Bal R, Wiig S. Exploring managers’ response to a quality and safety leadership intervention: findings from a multiple case study in Norwegian nursing homes and homecare services. BMJ Open Qual. 2021;10(3):e001494. 10.1136/bmjoq-2021-001494. PMC8354257 34373250

[63] Seljemo C, Viksveen P, Ree E. The role of transformational leadership, job demands and job resources for patient safety culture in Norwegian nursing homes: a cross-sectional study. BMC Health Serv Res. 2020;20(1):799. 10.1186/s12913-020-05671-y. PMC7448317 32847598

