# Supplementary material for: Nurses’ experiences with assessing pain in patients with dementia in nursing homes: a qualitative study
Source: BMC Nurs. 2026 Jul 27;25:1005. doi: 10.1186/s12912-026-05110-z (PMC13689034; doi:10.1186/s12912-026-05110-z)
Supplement: Supplementary file 1 [file 12912_2026_5110_MOESM1_ESM.docx]

**Interview guide – Individual semi-structured interviews with nurses in nursing homes**

The purpose is to collect data from nurses working in nursing homes about their experiences in assessing pain in patients with dementia.

**Introduction by moderator**

- Moderator and co-moderator present themselves and their roles during the interview.
- The participant presents themselves by name, education, seniority at the nursing home and experience in dementia care before the audio recording begins.
- Presentation of the topic and the aim of the study, as well as the participant’s rights.
- Information about anonymisation of the data material and that the participant could withdraw their consent at any time without questions asked.
- Information about internal and external confidentiality obligations, and what information that can be shared in the interview.
- Information that the interview will extend no further than an hour, and that there are no right or wrong answers.

**The main topic: Assessment of pain in patients with dementia**

- Can you tell us about your experiences assessing pain among nursing home patients with dementia?

Examples of follow-up prompts:

- You mentioned …, can you tell us a bit more about this?
- Can you explain your experiences in more detail?
- Can you elaborate on what you said about …
- What experiences have you had in assessing pain in patients with dementia?
- Do you believe that sufficient attention is given to assessing pain in patients with dementia?
- Can you explain how you assess pain in patients with dementia?
- Do you have any thoughts about other ways to assess pain in patients with dementia?
- Do you have any thoughts about what can increase the chances of effective and accurate pain assessment in this group of patients?
- Regarding pain assessment in patients with dementia, do you have any experiences that you find important to include in this study and that have not been mentioned yet?

**Summary**

Co-moderator summarise the collected data and ask whether the participant would like to add or elaborate on anything further.
